# A change of IL-2 and IL-4 production in patients with Helicobactor pylori infection

**DOI:** 10.1155/S0962935195000469

**Published:** 1995-07

**Authors:** X. G. Fan, J. Yakoob, X. J. Fan, P. W. N. Keeling

**Affiliations:** 1 Department of Infectious Diseases Hsiangya Hospital Hunan Medical University Hunan Changsha China

## Abstract

Hellcobacter pylori is the most common cause of gastroduodenal inflammation. However, the exact immune pathogenesis is not fully understood. To look for evidence of the immunological mechanism in H. pylori associated disease, we measured cytokine interleukin-2 (IL-2) and IL-4 levels produced by peripheral blood lymphocytes (PBL) and gastric biopsies in 20 subjects with or without H. pylori infection. H. pylori can stimulate IL-2 and IL-4 production from PBL in H. pylori negative as well as H. pylori positive individuals. The spontaneous IL-2 production by PBL and gastric biopsies was greater (p < 0.0025, <0.001)in  H. pylori negative individuals than that in H. pylori infected patients. Increased IL-4 levels from PBL in H. pylori infected patients were found in the presence of H. pylori (p < 0.0025). An increased spontaneous production of IL-4 from gastric biopsies was also observed in H. pylori infected patients (p < 0.025). In conclusion, an enhanced type 2 cytokine production was observed in H. pylori infected patients, which may be responsible for H. pylori chronic infection.


Mediators of Inflammation 4, 289–292 (1995)

### A change of IL-2 and IL-4 production in patients with *Helicobacter pylori* infection

X. G. Fan,[CA] J. Yakoob, X. J. Fan and P. W. N. Keeling

Department of Infectious Diseases, Hsiangya Hospital, Hunan Medical University, Changsha, Hunan, People's Republic of China

[CA]Corresponding Author

**HELICOBACTER *pylori* is the most common cause of gastroduodenal inflammation. However, the exact immune pathogenesis is not fully understood. To look for evidence of the immunological mechanism in *H. pylori* associated disease, we measured cytokine interleukin-2 (IL-2) and IL-4 levels produced by peripheral blood lymphocytes (PBL) and gastric biopsies in 20 subjects with or without *H. pylori* infection. *H. pylori* can stimulate IL-2 and IL-4 production from PBL in *H. pylori* negative as well as *H. pylori* positive individuals. The spontaneous IL-2 production by PBL and gastric biopsies was greater ($p < 0.0025$, $< 0.001$) in *H. pylori* negative individuals than that in *H. pylori* infected patients. Increased IL-4 levels from PBL in *H. pylori* infected patients were found in the presence of *H. pylori* ($p < 0.0025$). An increased spontaneous production of IL-4 from gastric biopsies was also observed in *H. pylori* infected patients ($p < 0.025$). In conclusion, an enhanced type 2 cytokine production was observed in *H. pylori* infected patients, which may be responsible for *H. pylori* chronic infection.**

**Key words:** *Helicobacter pylori*, Interleukin-2, Interleukin-4.

### Introduction

*Helicobacter pylori* are Gram-negative bacteria that live in the human stomach. *H. pylori* infection is now recognized as one of the most common chronic infections in man. Several lines of evidence implicate *H. pylori* infection in gastroduodenal inflammation.[1–3] Colonization of the gastric epithelium by the bacterium results in an inflammatory reaction by both the humoral and cellular immune responses.[4] In spite of some established observations, the exact immune factors that contribute to pathogenetic mechanisms of the disease and that sustain the chronic colonization are not fully understood.

T-helper (TH) lymphocytes may be subdivided into TH1 and TH2 subsets based on the distinct profiles of cytokine production. TH1 cells produce interferon-gamma (IFN-γ) and interleukin-2 (IL-2) which are associated with immunity or resistance to infection; whereas TH2 cells produce IL-4, IL-5, IL-6 and IL-10 cytokines which are associated with progression or persistence of infection. Type 1 cytokines stimulate cell-mediated immunity and type 2 cytokines stimulate humoral immunity. Cytokines produced by one type of TH cells can down-regulate the other type of TH cells. Enhanced TH2 reaction, which has an immunosuppressive role in human infection, has been found.[5–8] To evaluate the responsive conditions of TH1 and TH2 cells in subjects with *H. pylori* infection, we measured IL-2 produced by TH1 cells and IL-4 produced by TH2 cells in supernatants from peripheral blood lymphocytes (PBL) and gastric biopsies in these subjects.

### Subjects and Methods

*Subjects:* Twenty subjects with dyspepsia attending for upper gastrointestinal endoscopy were studied. None of the subjects had recently received non-steroidal anti-inflammatory drugs, bismuth compounds or antibiotics. Patients with evidence of malignant disease or immunosuppression were excluded. A rapid urease test (CLO-test, Delta West Ltd, Australia) and a histological examination by using a Giemsa stain were applied for evaluating the status of *H. pylori* infection. Subjects were designated as *H. pylori* positive on the basis of CLO testing or histological examination by Giemsa stain. Of 20 subjects, ten (seven men and three women; mean age 44.6 years, range 20–70) had *H. pylori* infection, including six with duodenal ulcer and four with gastritis alone. Ten (six men and four women; mean age 46.8 years, range 22–67) were *H. pylori* negative subjects with normal results of gastrointestinal endoscopy and histology.

© 1995 Rapid Science Publishers

*H. pylori: H. pylori* preparation was made by sonicating a mixture of *H. pylori* bacterial cultures obtained from six patients (four with duodenal ulcer and two with gastritis alone) on ice using 6 × 15 s, 100 watt pulses, with 30 s cooling intervals, followed by irradiation at 4000 rads to sterilize the preparation. It was then washed with phosphate buffered saline (PBS) and stored at −20°C.[3,9]

*PBL: PBL* were isolated by Ficoll–Hypaque density-gradient centrifugation, washed and suspended in RPMI 1640 medium containing 100 U/ml penicillin, 100 μg/ml streptomycin, 20 mM L-glutamine and 10% fetal calf serum. The number and viability of the isolated PBL were determined using acridine orange/ethidium bromide (EB/AO). PBL (2 × $10^5$/well) were seeded into each well of the 96-well plates. Duplicate cultures were incubated with *H. pylori* (final concentration 30 μg/ml), phytohaemagglutinin (PHA, 10 μg/ml as positive control) or the medium alone, respectively, at 37°C in 5% $CO_2$. After 72 h culture, supernatants were collected and stored at −70°C until cytokine assay.

*Gastric biopsies:* Multiple biopsy specimens were obtained during upper gastrointestinal endoscopy from adjacent sites of the gastric antrum for *H. pylori* histological examination, CLO-test and incubation. Biopsies for incubation were immediately washed twice with the medium and then cultured in 1 ml of culture medium at 37°C in 5% $CO_2$. Following a 24 h incubation, supernatants were collected by centrifugation and stored at −70°C until cytokine assay.[10]

*Measurements of IL-2 and IL-4:* IL-2 and IL-4 were measured by EIA Kits (Advanced Magnetics Inc., Cambridge, MA, USA). Assays were performed in duplicate according to the manufacturer's instructions.

*Statistics:* Data were expressed as medians and ranges. Statistical analysis was carried out using Mann–Whitney *U*-test.

### Results

*IL-2 levels: H. pylori* negative subjects had a significantly higher IL-2 level in supernatants from resting PBL compared with those having *H. pylori* infection. However, there was no significant difference between these two groups in IL-2 levels of supernatants from PBL, which were stimulated with PHA or *H. pylori* although an increase in IL-2 production was found following PHA or *H. pylori* stimulation in both of the two groups (Table 1).

There was decreased IL-2 production in supernatants from cultured gastric biopsies in patients with *H. pylori* infection when compared with subjects without (Table 2).

*IL-4 levels:* In the absence of PHA or *H. pylori*, no significant difference was found for IL-4 levels in supernatants from cultured PBL between *H. pylori* negative and positive subjects. Stimulation

Table 1. IL-2 levels in supernatants from lymphocytes (pg/ml)

| | IL-2 | | |
|---|---|---|---|
| | Spontaneous | PHA | *H. pylori* |
| *H. pylori* negative subjects | | | |
| Median | 19.80* | 102.9 | 30.38 |
| Range | 11.96–25.52 | 58.32–155.3 | 20.75–47.24 |
| No. | 10 | 10 | 10 |
| *H. pylori* positive subjects | | | |
| Median | 14.38 | 104.7 | 30.66 |
| Range | 10.12–21.18 | 50.68–162.4 | 18.95–45.25 |
| No. | 10 | 10 | 10 |

*$p < 0.0025$ *vs. H. pylori* positive subjects.

Table 2. IL-2 and IL-4 production in supernatants from cultured gastric biopsies (pg/g wet wt)

| | IL-2 ($n$ = 10) | IL-4 ($n$ = 10) |
|---|---|---|
| *H. pylori* negative subjects | | |
| Median | 635.1* | 47.91** |
| Range | 359.1–1368 | 16.24–67.28 |
| *H. pylori* positive subjects | | |
| Median | 337.3 | 67.57 |
| Range | 141.8–576.8 | 28.28–101.3 |

*$p < 0.0025$ or **$p < 0.025$ *vs. H. pylori* positive subjects, respectively.

Table 3. IL-4 level in supernatants from lymphocytes (pg/ml)

| | IL-4 | | |
|---|---|---|---|
| | Spontaneous | PHA | *H. pylori* |
| *H. pylori* negative subjects | | | |
| Median | 10.87 | 44.0 | 21.71* |
| Range | 5.92–15.24 | 19.92–60.65 | 18.81–38.40 |
| No. | 10 | 10 | 10 |
| *H. pylori* positive subjects | | | |
| Median | 11.86 | 43.36 | 30.23 |
| Range | 6.48–17.48 | 23.30–64.90 | 23.77–42.18 |
| No. | 10 | 10 | 10 |

*$p < 0.0025$ *vs. H. pylori* positive subjects.

with *H. pylori* resulted in higher production of IL-4 from PBL in *H. pylori* infected patients than that in *H. pylori* negative individuals. Although an increase in IL-4 production was observed in supernatants from PHA activated PBL in all subjects, IL-4 production was not significantly different between the two groups (Table 3).

A significantly higher level of IL-4 in supernatants from cultured gastric biopsies was observed in *H. pylori* infected patients compared to *H. pylori* negative subjects (Table 2).

### Discussion

Understanding host responses to infection requires knowledge of the regulatory mechanisms that affect the outcome of infection by the pathogen. The majority of individuals exposed develop a powerful cellular immune response to the pathogen, which results in elimination of the infection. Some individuals are unable to mount a sufficient immune response so that the infection proceeds unabated.[11] A few studies have reported that IL-4 has negative immunoregulatory function in some infectious diseases, such as acquired immunodeficiency syndrome, leprosy and tuberculosis.[5,6,12] Enhanced TH2 reaction and down-regulation of the cellular immune responses have been proposed in *H. pylori* infected patients.[11,13] The study showed that increased IL-4 and decreased IL-2 production was observed in PBL as well as in gastric biopsies from *H. pylori* infected patients, providing experimental evidence that enhanced TH2 reaction is present in *H. pylori* infected patients and may play an important role in the immune pathogenesis of *H. pylori* infection.

Microbial and host factors that affect the clearance of *H. pylori* from gastric tissue have not been fully identified. Despite the fact that hosts with *H. pylori* infection may have immune or inflammatory responses to *H. pylori*, the disease still persists.[1,2] A TH1 to TH2 reaction shift may be one of several important factors in this situation.[11] Although the majority of people colonized with *H. pylori* elicit strong, specific circulating and gastric mucosal antibody responses, the humoral response seems ineffective at eradicating *H. pylori*.[14,15] Suppressed TH1 and enhanced TH2 responses in *H. pylori* infection may imply that the human host attempts to down-regulate the inflammatory responses and reduce the tissue injury because the intense inflammatory responses for the host *per se* are destructive. Some hosts, on the other hand, are unable to eliminate the bacterium due to suppressed cellular immune responses. This may be one possible explanation why *H. pylori* infection is a chronic condition, possibly lasting for decades, if not for life.

It has been demonstrated previously that *H. pylori* can activate PBL to release inflammatory cytokines.[16,17] Also, a higher gastric cytokine concentration is present in *H. pylori* positive patients.[18] In the present data, although PHA can stimulate cytokine production from PBL, no significant difference was found between *H. pylori* positive and negative groups in IL-4 production, suggesting this may be a specific response to *H. pylori* for *H. pylori* infected hosts. It has been known that IL-4 can inhibit the synthesis of IFN-γ (type 1 cytokine).[19,20] Suppressed proliferative responses of peripheral blood and gastric lymphocytes and decreased production of IFN-γ from PBL and gastric lamina propria lymphocytes have also been demonstrated in patients with *H. pylori* colonization,[16,21,22] implying that specific T-cell responses are down-regulated by type 2 cytokines, and enhanced IL-4 profile is responsible for decreased production of IFN-γ in *H. pylori* infection. Further experimental observations will be required to investigate whether the balance between TH1 and TH2 responses would be restored by use of recombinant type 1 cytokines and anti-type 2 cytokine antibodies, or following eradication of *H. pylori* in the infected patients.

More fundamentally, treatment of *H. pylori* is difficult because *H. pylori*'s habitat below the layer of gastric mucus hinders the necessary access of antimicrobials. Also, bacterial resistance to antimicrobials is the main problem with current treatment regimes.[23] Thus, antibacterial treatment and effective immune responses will both be of importance in the eradication of *H. pylori*. We might be able to induce proper immune responses in *H. pylori* positive hosts and ultimately eradicate the bacterium. We might use some agents to reduce the overproduction of type 2 cytokines or to favour the secretion of type 1 cytokines. The administration of recombinant type 1 cytokines could be beneficial to *H. pylori* infected hosts. Anti-type 2 cytokine antibodies or type 2 cytokine receptor antagonists could be used to restore the balance between TH1 and TH2 responses in such patients and thus eliminate the organism.

### References

1. Blaser MJ. *Helicobacter pylori*: microbiology of a 'slow' bacterial infection. *Trends in Microbiology* 1993; **1:** 255–260.
2. Moss S, Calam J. *Helicobacter pylori* and peptic ulcers: the present position. *Gut* 1992; **33:** 289–292.
3. Fan XG, Chua A, Fan XJ, Keeling PWN. Lipid peroxidation induced by *Helicobacter pylori*. *Exp Clin Gastroenterol* 1994; **4:** 29–34.

4. Ernst PB, Navarro JJ, Reyes V, Crowe S. Overview of the immune response to *H. pylori*. In: Hunt RH, Tytgat GNT, eds. Helicobacter pylori: *Basic Mechanisms to Clinical Cure*. Lancaster, UK: Kluwer Academic Publishers, 1994; 295–305.
5. Modlin RL, Nutman TB. Type 2 cytokines and negative immune regulation in human infections. *Current Opin Immunol* 1993; **5:** 511–517.
6. Clerici M, Shearer GM. A TH1-TH2 switch is a critical step in the etiology of HIV infection. *Immunol Today* 1993; **14:** 107–111.
7. Fiorentino DF, Bond MW, Mosmann TR. Two types of mouse T helper cell, IV: Th2 clones secrete a factor that inhibits cytokine production by Th1 clones. *J Exp Med* 1989; **170:** 2081–2098.
8. Martinez OM, Gibbons RS, Garovoy MR, Aronson FR. IL-4 inhibits IL-2 receptor expression and IL-2-dependent proliferation of human T cells. *J Immunol* 1990; **144:** 2211–2215.
9. Brennan DP, Keeling PWN. *Campylobacter pylori* released membrane-associated and soluble antigens during infection. In: Megraud F, *et al*, eds. *Gastroduodenal Pathology and* Campylobacter pylori. Amsterdam: Elsevier Sci. Pub. B.V. 1989; 207–211.
10. Wardle TD, Hall L, Turnberg LA. Inter-relationships between inflammatory mediators released from colonic mucosa in ulcerative colitis and their effects on colonic secretion. *Gut* 1993; **34:** 503–508.
11. Blaser MJ, Parsonnet J. Parasitism by the 'slow' bacterium *Helicobacter pylori* leads to altered gastric homeostasis and neoplasia. *J Clin Invest* 1994; **94:** 4–8.
12. Sieling PA, Abrams JS, Yamamura M, *et al*. Immunosuppressive roles for IL-10 and IL-4 in human infection. *In vitro* modulation of T cell responses in leprosy. *J Immunol* 1993; **150:** 5501–5510.
13. Croitoru K. Down-regulation of the immune response to *H. pylori*. In: Hunt RH, Tytgat GNT, eds. Helicobacter pylori: *Basic Mechanisms to Clinical Cure*. Lancaster, UK: Kluwer Academic Publishers, 1994; 333–341.
14. Newell DG, Stacey AR. B cell responses in *H. pylori* infection. In: Hunt RH, Tytgat GNT, eds. Helicobacter pylori: *Basic Mechanisms to Clinical Cure*. Lancaster, UK: Kluwer Academic Publishers, 1994; 309–320.
15. Marshall BJ. Helicobacter pylori. *Am J Gastroenterol* 1994; **89:** s116–s128.
16. Fan XJ, Chua A, Shahi CN, McDevitt J, Keeling PWN, Kelleher D. Gastric T lymphocyte responses to *Helicobacter pylori* in patients with *H. pylori* colonisation. *Gut* 1994; **35:** 1379–1384.
17. Fan XJ, Chua A, O'Connell M, Kelleher D, Keeling PWN. Interferon-gamma and tumor necrosis factor production in patients with *Helicobacter pylori* infection. *Ir J Med Sci* 1993; **162:** 408–411.
18. Fan XG, Chua A, Fan XJ, Keeling PWN. Increased gastric production of interleukin-8 and tumour necrosis factor in patients with *Helicobacter pylori* infection. *J Clin Pathol* 1995; **48:** 133–136.
19. Erard F, Wild MT, Garcia-Sanz JA, Gros GL. Switch of CD8 T cells to noncytolytic CD8-CD4- cells that make TH2 cytokines and help B cells. *Science* 1993; **260:** 1802–1805.
20. Danzer SG, Kirchner H, Rink L. Cytokine interactions in human mixed lymphocyte culture. *Transplantation* 1994; **57:** 1638–1642.
21. Karttunen R, Andersson G, Poikonen K, Kosunen TU, Karttunen T, Juutinen K. *Helicobacter pylori* induces lymphocyte activation in peripheral blood cultures. *Clin Exp Immunol* 1990; **82:** 485–488.
22. Karttunen R. Blood lymphocyte proliferation, cytokine secretion and appearance of T cells with activation surface markers in cultures with *Helicobacter pylori*. Comparison of the responses of subjects with and without antibodies to *H. pylori*. *Clin Exp Immunol* 1991; **83:** 396–400.
23. Tytgat GNJ, Lee A, Graham DY, Dixon MF, Rokkas T. The role of infectious agents in peptic ulcer disease. *Gastroenterol Internat* 1993; **6:** 76–89.

ACKNOWLEDGEMENT. We are indebted to Dr H. X. Xia of the Dept. of Clinical Microbiology, St James's Hospital, Dublin, for kindly providing *H. pylori*.

**Received 26 April 1995;**
**Accepted 1 May 1995**
